# Energy deficiency promotes rhythmic foraging behavior by activating neurons in paraventricular hypothalamic nucleus

**DOI:** 10.3389/fnut.2023.1278906

**Published:** 2023-10-12

**Authors:** Shanshan Wu, Jing Wang, Yang Xu, Zicheng Zhang, Xinchen Jin, Yixiao Liang, Yueping Ge, Huidong Zhan, Li Peng, Dandan Luo, Mengzhu Li, Wenkai Bi, Qingbo Guan, Zhao He

**Affiliations:** ^1^Department of Endocrinology, Shandong Provincial Hospital & Medical Integration and Practice Center, Shandong University, Jinan, Shandong, China; ^2^Key Laboratory of Endocrine Glucose & Lipids Metabolism and Brain Aging, Ministry of Education, Shandong Provincial Hospital Affiliated to Shandong First Medical University, Jinan, Shandong, China; ^3^Shandong Key Laboratory of Endocrinology and Lipid Metabolism, Shandong Institute of Endocrine and Metabolic Diseases, Shandong Clinical Research Center of Diabetes and Metabolic Diseases, Shandong Prevention and Control Engineering Laboratory of Endocrine and Metabolic Diseases, Shandong Provincial Hospital Affiliated to Shandong First Medical University, Jinan, Shandong, China; ^4^Key Laboratory of Cardiovascular Remodeling and Function Research, Chinese Ministry of Education, Chinese National Health Commission and Chinese Academy of Medical Sciences, The State and Shandong Province Joint Key Laboratory of Translational Cardiovascular Medicine, Department of Cardiology, Qilu Hospital, Cheeloo College of Medicine, Shandong University, Jinan, Shandong, China; ^5^School of Information Management, Nanjing University, Nanjing, Jiangsu, China; ^6^Advanced Medical Research Institute, Cheeloo College of Medicine, Shandong University, Jinan, Shandong, China

**Keywords:** hanging behavior, food cues, CNO, nutrients, brain regions

## Abstract

**Background:**

Dysregulation of feeding behavior leads to a variety of pathological manifestations ranging from obesity to anorexia. The foraging behavior of animals affected by food deficiency is not fully understood.

**Methods:**

Home-Cage system was used to monitor the behaviors. Immunohistochemical staining was used to monitor the trend of neuronal activity. Chemogenetic approach was used to modify neuronal activity.

**Results:**

We described here a unique mouse model of foraging behavior and unveiled that food deprivation significantly increases the general activities of mice with a daily rhythmic pattern, particularly foraging behavior. The increased foraging behavior is potentiated by food cues (mouthfeel, odor, size, and shape) and energy deficit, rather than macronutrient protein, carbohydrate, and fat. Notably, energy deficiency increases nocturnal neuronal activity in paraventricular hypothalamic nucleus (PVH), accompanying a similar change in rhythmic foraging behavior. Activating neuronal activity in PVH enhances the amplitude of foraging behavior in mice. Conversely, inactivating neuronal activity in PVH decreases the amplitude of foraging behavior and impairs the rhythm of foraging behavior.

**Discussion:**

These results illustrate that energy status and food cues regulate the rhythmic foraging behavior via PVH neuronal activity. Understanding foraging behavior provides insights into the underlying mechanism of eating-related disorders.

## Introduction

Free-eating animals exhibit a strong circadian rhythm in their feeding behavior, particularly foraging behavior (1). Foraging behavior is a fundamental physiological activity of animal survival for obtaining necessary energy. During the active phase, restricting food increases the general activities of mice, particularly wakefulness and foraging behavior (2). Consistently, brain regions associated with reward and motivation are activated during the status of energy deficit, which enhances the salience of food-related stimuli and increases the motivation to forage (3, 4).

Energy shortage is customarily recognized as the main cause of food deprivation-promoted foraging behavior. Energy mainly derives from three types of nutrients in food: carbohydrate, protein, and fat, which have been associated with the regulation of animal appetite and food intake (5). Increasing evidence has shown that food nutrients regulate animal foraging behavior. For example, high-protein and high-carbohydrate foods increased foraging behavior in herbivorous mice (6). In contrast, high-protein and high-carbohydrate foods decrease foraging behavior in omnivorous and carnivorous mice (6), and chimpanzees prefer to forage for leaves with a high carbohydrate content (7). Furthermore, foods rich in fat are more likely to increase foraging behavior in rodents (8). In addition, food cues, such as olfactory, visual signals, and mouthfeel, can increase the eating-related behavior of mice even in the absence of energy depletion (4, 9). For example, high food density reduces foraging behavior (10) and the sense of smell improves foraging efficiency (11). However, it is unknown the roles of food cues and macronutrients in the increased foraging behavior elicited by food deprivation.

The periventricular hypothalamus (PVH) is a vital brain region involved in regulating appetite and eating behavior. Appetite-related neuropeptide Y (NPY) injection to PVH decreases foraging behavior but increases hoarding and food intake (12). Moreover, melanocortin 4 receptor (MC4R)-expressing neurons in PVH regulate feeding behavior (13–16), as evidenced by food deprivation increases the activity of PVH^MC4R^ neurons *via* inhibiting GABA release (17). In addition, different subpopulations of neurons in PVH control distinguishing aspects of energy balance. For example, MC4R neurons in PVH modulate the satiety by projecting to the hindbrain (18, 19). Neuronal nitric oxide synthase 1 (*Nos1*) neurons in PVH control both feeding behavior and energy expenditure (20). In contrast, parvocellular OXT neurons are a subpopulation of the PVH^Nos1^ field, which modestly participates in the control of energy expenditure, rather than feeding (21, 22). Taken together, the periventricular hypothalamus acts as a central orchestrator in the intricate process of eating behavior regulation.

In this paper, to investigate the nature of the foraging behavior of caged mice, we use the Home-Cage system to monitor the fine behaviors of fasted mice in real-time and disclose the intrinsic neural regulatory mechanisms.

## Materials and methods

### Animals

Eight weeks C57BL/6 J male mice (Charles River Laboratory, Beijing, China) were kept under standard conditions with temperature (22 ± 1°C) and humidity (~40%) in a 12 h light/12 h dark cycle, free access to food and water. Mice were used for experiments at the age of 10–12 weeks. All of mice subjected to invasive experiments were used once. All animal experiments were permitted by the Institutional Animal Care and Use Committee at Shandong Provincial Hospital and complied with the China National Regulations on the Administration of Experimental or Laboratory Animals (No.2, 20,170,301, SSTC, China), and the ARRIVE guidelines or the U.K. Animals (Scientific Procedures) Act, 1986.

### Gel diet

The gel diets were purchased from Jiangsu Xietong (Nanjing, China). Experiments were performed after 2 days of acclimation with full-energy gel. The ingredients of different gels were listed as follows: see Table 1.

**Table 1:** Ingredients of different gels.

g/kcal	FEG	NEG	PFG	FFG	CFG
Colloidal premix	13.7/0	13.7/0	13.7/0	13.7/0	13.7/0
ddH_2_O_2_	737.4/0	962.44/0	743.4/0	715.4/0	775.4/0
Casein	50/200	0/0	0/0	59.5/238	138.13/552.52
L-Cystine	0.75/3	0/0	0/0	0.75/3	0.75/3
Com Starch	13.5/54	0/0	13.5/54	13.5/54	0/0
Maltodextrin 39	13/52	0/0	13/52	13/52	0/0
Sucrose	130/520	0/0	170.5/682	159.9/639.6	0/0
Cellulose	12.5/0	12.5/0	12.5/0	12.5/0	12.5/0
Olive oil	17.5/157.5	0/0	22/198	0/0	47.89/431.01
Vitamin mix V10037	2.5/10	0/0	2.5/10	2.5/10	2.5/10
Mineral mix S10022G	8.75/0	0/0	8.75/0	8.75/0	8.75/0
Choline Bitartrate	0.63/0	0/0	0.63/0	0.63/0	0.63/0
Total	1000/996.5	1001/0	1000/996	1000/996.6	1000/996.53

Ingredients of different gels and mass (g)/energy (kcal) in each kilogram of gel. FEG, full-energy gel; NEG, none-energy gel; PFG, protein-free gel; FFG, fat-free gel; CFG, carbohydrate-free gel.

### Home-cage monitoring

Mice were kept in individual cages for home behavior monitoring and analysis by Home-Cage system (Shanghai Vanbi Intelligent Technology Co., Ltd.) on a 12 h light/12 h dark cycle and an ambient temperature at 24 ± 2°C in a silent room, with fluorescent light to simulate daytime and light off to simulate night, without disrupting the normal circadian rhythm of the mice. Mice were monitored by an infrared camera (Shanghai Vanbi Intelligent Technology Co., Ltd.) in the Home-Cage box mounted horizontally on the side of the cage for 24 h. The videos captured by these cameras are stored in a video processor connected to the computer. Video data were analyzed by Tracking Master V3.1.56 software (Shanghai Vanbi Intelligent Technology Co., Ltd.), and behavioral definitions were as described previously (23). Tracking Master V3.1.56 software monitored parameters as follows: contour erosin (2 pixels), contour expansion (2 pixels), animal size (500–10,000 pixels), motionless (0–4 cm/s), active (> = 4 cm/s), slow active (4–10 cm/s), fast active (> = 20 cm/s), move between two frames (1–10,000 pixels).

For the detection of home behavior in mice fed with NEG, FEG, PFG, FFG, and CFG, 2 days of FEG feeding habituation were conducted before Home-Cage monitoring. For the detection of home behaviors in virus-injected mice, 3 weeks after virus injection, a single intraperitoneal injection of clozapine-n-oxide (CNO, 1 mg/kg) was performed 5 min before the start of the experiment, and Home-Cage monitoring was performed 5 min later.

### Energy intake

Energy intake in mice was performed with the Home-Cage experiment simultaneously. The weight of gels (before) was recorded at the start of and the end of the 24 h monitoring (after), respectively. Energy intake = (before-after) * 0.996/body weight. 0.996 is calories per gram of gels.

### Immunohistochemical staining

Deeply anesthetized mice were perfused with 4% paraformaldehyde, and the brains were removed to the same fixative for 0–14 h. Using an automatic vibrating slicer (Leica, VT1200S), 30-um-thick brain sections were collected. Brain sections were incubated with c-fos antibody (1:500, Abcam, ab214672) and visualized with the 3,3′-diaminobenzidine (DAB) chromogen kit (DAB kit, Zhongshan Golden Bridge Bio-technology Co.Ltd). Images were acquired by positive microscope (Karl Zeiss A2, Germany). C-fos positive cells were counted in DMH (Bregma −1.70 mm), PVH (Bregma −0.94 mm), ARC (Bregma −1.70 mm), POA (Bregma 0.5 mm), VMH (Bregma −1.70 mm), LH (Bregma −1.70 mm) and SCN (Bregma −0.46 mm) by manual counting. The area (mm^2^) of each region of hypothalamus was calculated by image J. The density of c-fos positive cells = the number of c-fos positive cells / the area.

### Viruses information and stereotaxic surgeries

Modulation of neuronal activity using Designer Receptors Exclusively Activated by Designer Drugs (DREADDs), a technology that allows for repeated activation of engineered receptors by systemic injection of otherwise inert ligand clozapine N-oxide (CNO) (24). By adeno-associated virus (AAV) as vectors, the inhibitory hM4D(Gi) DREADDs (pAAV2/9-hSyn-hM4D(Gi)-mCherry-WPRE, ObiO, AG50475, 1E+ 12 GC/ml) was used to inhibit neuronal activity, the activated DREADDs hM3D(Gq) (pAAV2/9-hSyn-hM3D(Gq)-EGFP-WPRE, ObiO, H6568, 1E+ 12 GC/ml) were used to activate neural activity. The empty control vector was pAAV-SYN-MCS-EYFP-3FLAG (AOV067, 1E+ 12 GC/ml). These AAVs were purchased from ObiO Technology (Shanghai, China). Mice received intraperitoneal injections of CNO (1 mg/kg, Sigma, C0832-5MG) to activate DREADD receptors. The dosage was chosen based on prior work showing that 1 mg/kg is an effective dose (25).

Viruses were stereotaxically infused into PVH as previous report (26), mice were placed on a stereotaxic alignment system (1900, David Kopf Instruments), and anesthetization by 2% isoflurane was maintained during surgeries. The coordinate for AAV injection (0.3ul/side) about bregma according to Mouse Brain Atlas (George Paxinos and Keith B. J. Franklin, second edition) was AP −0.82 mm, ML ±0.2 mm, DV −4.75 mm (PVH). Only animals with the correct injection site were included in the study.

### Statistical analysis

Data were represented as mean ± SEM. An unpaired t-test was used to compare the means of two samples. One-way analysis of variance (ANOVA) was used to analyze and compare of means of three or more samples. Scheirer-Ray-Hare test was used to analyze sequential measurements. *p* < 0.05 was considered statistically significant. ANCOVA was performed with SPSS Statistics version 26 (IBM Corp.), the Scheirer-Ray-Hare test was performed with R software version 4.3.1, and the unpaired t-test was performed with Prism 9.0 (GraphPad Software Inc.).

## Results

**Figure 1:**
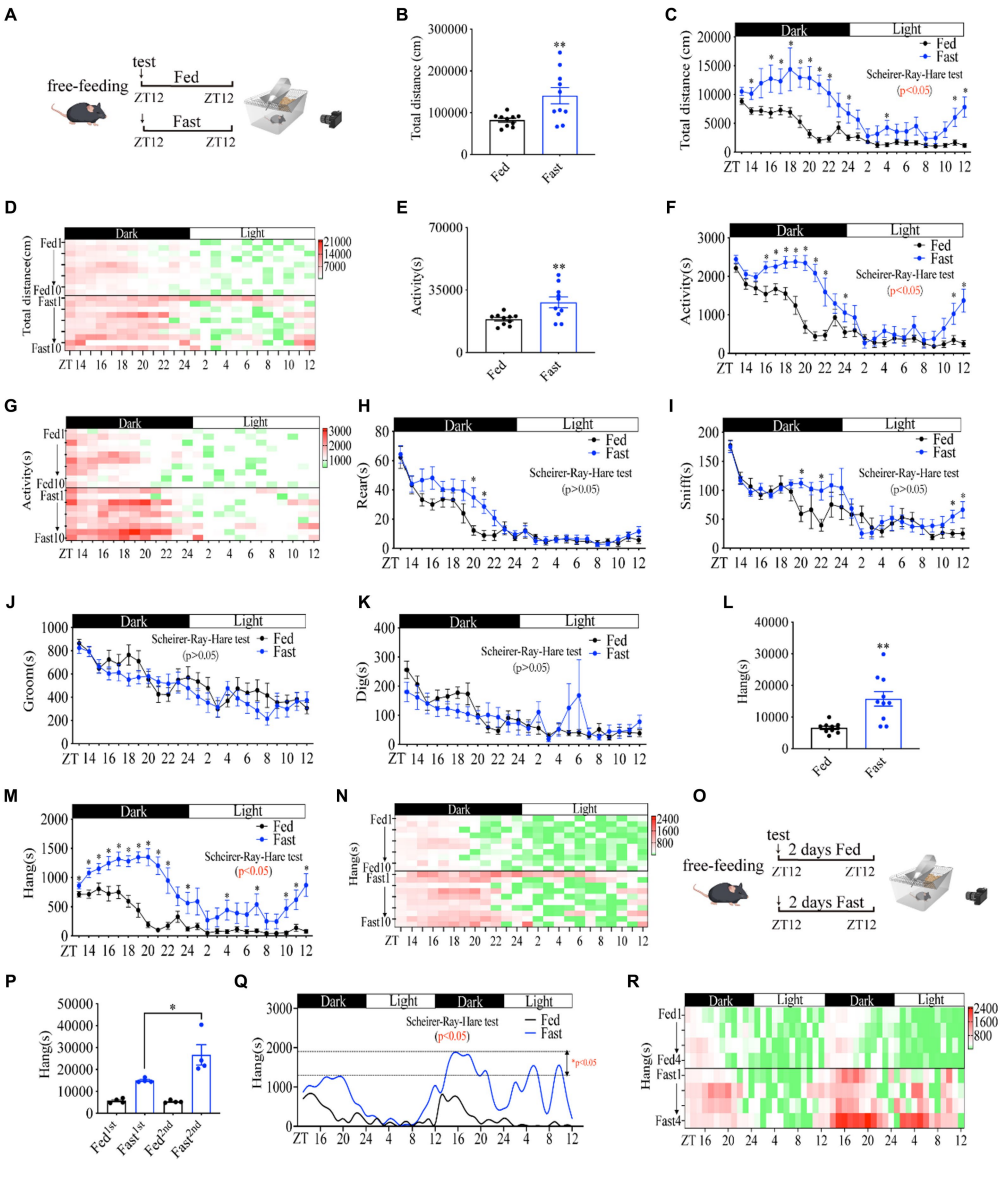
Food deprivation increases mouse foraging behavior in a rhythmic manner. **(A)** A diagram of experimental scheme (10 weeks C57BL/6 J male mice fed with chow diet or fasting, *n* = 10:10). **(B)** Total distance in 24 h. **(C)** Distance curve over 24 h. **(D)** Distance/h in 24 h for each mouse. **(E)** Total time of activity in 24 h. **(F)** Activity curve over 24 h. **(G)** Activity/h in 24 h for each mouse. **(H)** Rearing curve over 24 h. **(I)** Sniffing curve over 24 h. **(J)** Grooming curve over 24 h. **(K)** Digging curve over 24 h. **(L)** Total time of hanging in 24 h. **(M)** Hanging time curve over 24 h. **(N)** Hanging time/h in 24 h for each mouse. **(O)** A diagram of experimental scheme (10 weeks C57BL/6 J male mice fed with chow diet or fasting, *n* = 4:4). **(P)** Total time of hanging in 2 days. **(Q)** Hanging time curve over 2 days. **(R)** Hanging/h in 2 days for each mouse. Data are represented as mean ± SEM, ^*^p < 0.05, ^**^p < 0.01. Data in panels **B–L** were analyzed using an unpaired *t*-test, data in panel **P** was analyzed using One-way ANOVA, and other data were analyzed using the Scheirer-Ray-Hare test. ZT, zeitgeber time.

Free-feeding rodents exhibit strongly self-sustained properties in general activity with daily rhythms (27). To analyze mouse activities during the fasting period, we utilized Home-Cage system to monitor mice behaviors in real-time for 24 h under fasting or free-feeding conditions, respectively (Figure 1A). The mice were *ad libitum* access to water during the fasting experiment. Consistent with previous literature (28, 29), the total locomotor distance (Figures 1B–D) and general activity (Figures 1E–G) during the whole night were significantly increased in fasted mice as compared to fed mice. The rearing (Figure 1H and Supplementary Figures S1A,B) and sniffing (Figure 1I and Supplementary Figures S1C,D) behaviors of fasted mice were unaltered, even though sniffing and rearing were higher than fed mice at several time points (Figures 1H,I). Total grooming (Figure 1J and Supplementary Figures S1E,F), and digging (Figure 1K and Supplementary Figures S1G,H) were decreased. In contrast, hanging behavior was dramatically increased (Figures 1L–N) similar to total distance and general activity (Figures 1B–G). Considering the increased foraging behavior of mice under food deficiency (4, 30), we disclose that the hanging behavior of cage mice represents the foraging behavior.

To examine whether foraging behavior has the property of self-sustained rhythm, we performed a real-time monitor of fasted mice behaviors for 48 h (Figure 1O). Notably, the lowest amplitude of foraging behavior in fed mice was always observed in the daytime at the daily ZT2-8 and the highest amplitude of foraging behavior appeared in the night from ZT12-14, revealing an intrinsic oscillator in regulating foraging behavior (Figures 1Q,R). Mice in the second 24 h of fasting displayed a dramatically higher amplitude of foraging behavior than that of mice in the first 24 h of fasting (Figure 1P). In particular, the increase in amplitude in the second daytime of fasting was extremely higher than that of the first daytime (Figures 1Q,R), suggesting a critical regulatory role of fasting in foraging behavior intensity. Together, these data demonstrate that hanging behavior is a self-sustained daily rhythmic activity and is primarily regulated by fasting or hunger signals, which reflects the properties of foraging behavior.

**Figure 2:**
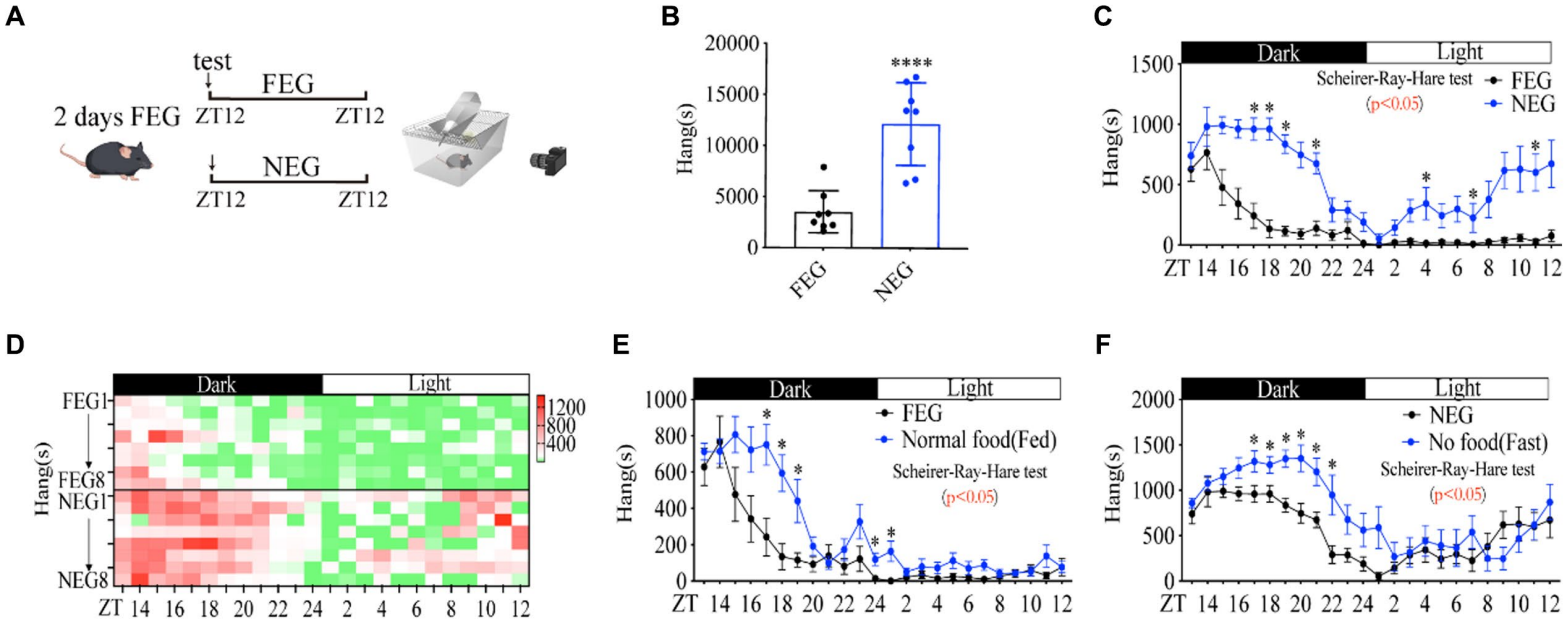
Food cues increase the amplitude of rhythmic foraging behavior. **(A)** A diagram of experimental scheme (12 weeks C57BL/6 J male mice fed with FEG or NEG, *n* = 8:8). **(B)** Total time of hanging in 24 h. **(C)** Hanging time curve over 24 h. **(D)** Hanging time/h in 24 h for each mouse. **(E)** Comparison of foraging behavior between FEG from **C** and normal diet from Figure 1M (*n* = 8:10). **(F)** Comparison of foraging behavior between NEG from **C** and fasted from Figure 1M (*n* = 8:10). Data are represented as mean ± SEM, ^*^p < 0.05, ^****^p < 0.0001. Data in panel **B** were analyzed using an unpaired *t*-test and other data were analyzed using the Scheirer-Ray-Hare test. FEG, full-energy gel; NEG, non-energy gel; ZT, zeitgeber time.

Food cues (mouthfeel, odor, size, and shape) are associated with the rewarding system, which is involved in the modulation of foraging behavior (31, 32). To examine whether the absence of food cues by food deprivation affects the increase in foraging behavior, we replaced the chow diet with gel diet when mice behaviors were monitored, all of mice were adapted to full energy gel (FEG) for another 2 days before this experiment (Figure 2A). As expected, none energy gel (NEG) dramatically induced a higher amplitude of distance (Supplementary Figures S2A–C), activity time (Supplementary Figures S2D–F) and foraging behavior (Figures 2B–D) than FEG did. In contrast, other behaviors of mice were not significantly altered (Supplementary Figures S2G–R), indicating a specific role of energy deficiency in foraging behavior. Of note, compared with fasted mice (Supplementary Figures S1E–H), mice fed with NEG showed unaltered grooming and digging behaviors (Supplementary Figures S2M–R), suggesting food cues were involved in regulating grooming and digging behaviors.

Notably, comparing the data between Figures 1M and Figures 2C, we found that FEG led to a lower amplitude of foraging behavior than normal chow diet (Figure 2E), and NEG also reduced the amplitude of foraging behavior as compared to fasting by food deprivation (Figure 2F), elucidating that food cues (mouthfeel, odor, size, and shape) are involved in promoting rhythmic foraging behavior.

**Figure 3:**
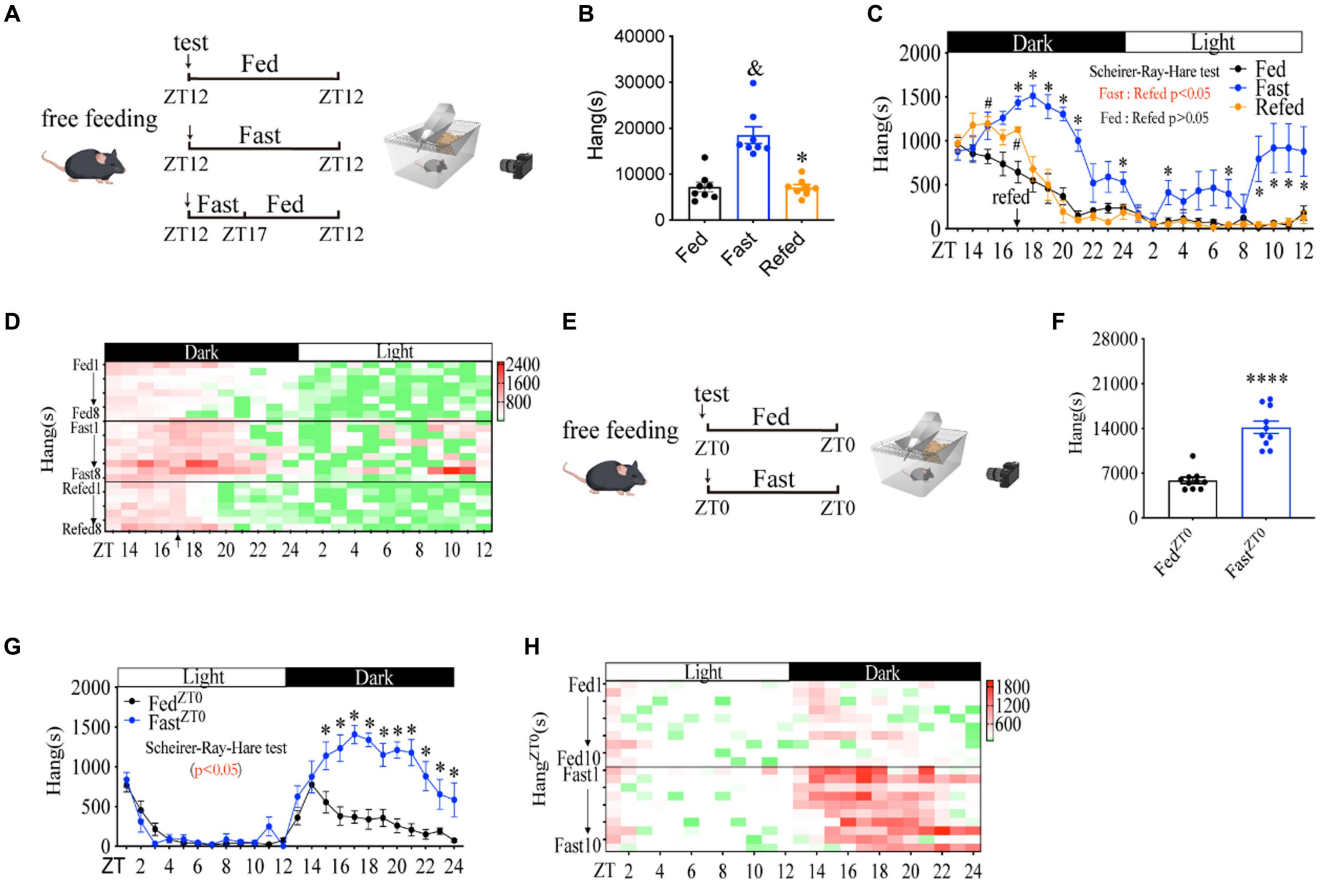
Habitual rhythm foraging behavior is dramatically increased by energy shortage. **(A)** A diagram of experimental scheme (10 weeks C57BL/6 J male mice were divided into three groups, *n* = 8:8:8). **(B)** Total time of hanging in 24 h. **(C)** Hanging time curve over 24 h. **(D)** Hanging time/h in 24 h for each mouse. **(E)** A diagram of experimental scheme (12 weeks C57BL/6 J male mice fed with chow diet or fasted, *n* = 10:10). **(F)** Total time of hanging in 24 h. **(G)** Hanging time curve over 24 h (started from ZT0), **(H)** hanging time/h in 24 h for each mouse(started from ZT0). Data are represented as mean ± SEM. In panels **B,C**, ^&^ represents the significance between fed and fast mice. ^#^ represents the significance between fed and fast mice. * represents the significance between fast and refed mice. In panels **F,G**, * represents the significance between fast and fast mice. ^&^*p* < 0.05, ^#^*p* < 0.05, ^*^p < 0.05, ^***^p < 0.001, ^****^p < 0.0001. Data in panel **B** were analyzed using One-way ANOVA, data in panel **F** were analyzed using an unpaired *t*-test, and other data were analyzed using the Scheirer-Ray-Hare test. ZT, zeitgeber time.

The calorie contents are potent natural reward and conditioning stimulus signals in the rewarding system (33). Indeed, fasting significantly increased the amplitude of foraging behavior, and subsequent refeeding after 5 h of fasting (Figure 3A) terminated the increase in distance (Supplementary Figures S3A–C), activity time (Supplementary Figures S3D–F) and foraging behavior of mice (Figures 3B–D). In contrast, rearing (Supplementary Figures S3E–G), sniffing (Supplementary Figures S3H–J), and grooming (Supplementary Figures S3K–M) in refed mice were not significantly altered compared to these in fed and fasted mice. Notably, refed mice exhibited reduced digging behavior compared to fed mice (Supplementary Figures S3N,O), which is consistent with the reduced digging behavior under food deprivation conditions (Supplementary Figures S1G, S3P).

Similarly, when mice were monitored from ZT0 (Figure 3E), we found that the distance (Supplementary Figures S4A–C), activity time (Supplementary Figures S4D–F), and foraging behavior (Figures 3F–H) in fasted mice were dramatically increased at night time. In contrast, fasted mice exhibited a similar foraging behavior as free-feeding mice within daytime ZT0-ZT12 (Figures 3G,H). The unchanged foraging behavior during the daytime was possible due to mice with sufficient energy supply after a full meal from the last night (34). Rearing (Supplementary Figures S4G–I) and sniffing (Supplementary Figures S4J–L) were not altered significantly (Scheirer-Ray-Hare test, *p* > 0.05), even though the total sniffing was decreased (Supplementary Figures S4J,M). Consistent with starting from 7 pm, grooming (Supplementary Figures S4M–O) also decreased and digging (Supplementary Figures S4P–R) behaviors had a decreased trend (Scheirer-Ray-Hare test, *p* < 0.05), even though the total digging time was not altered significantly. These results demonstrate that the increase of habitually rhythmic foraging behavior induced by fasting is primarily caused by energy shortage.

**Figure 4:**
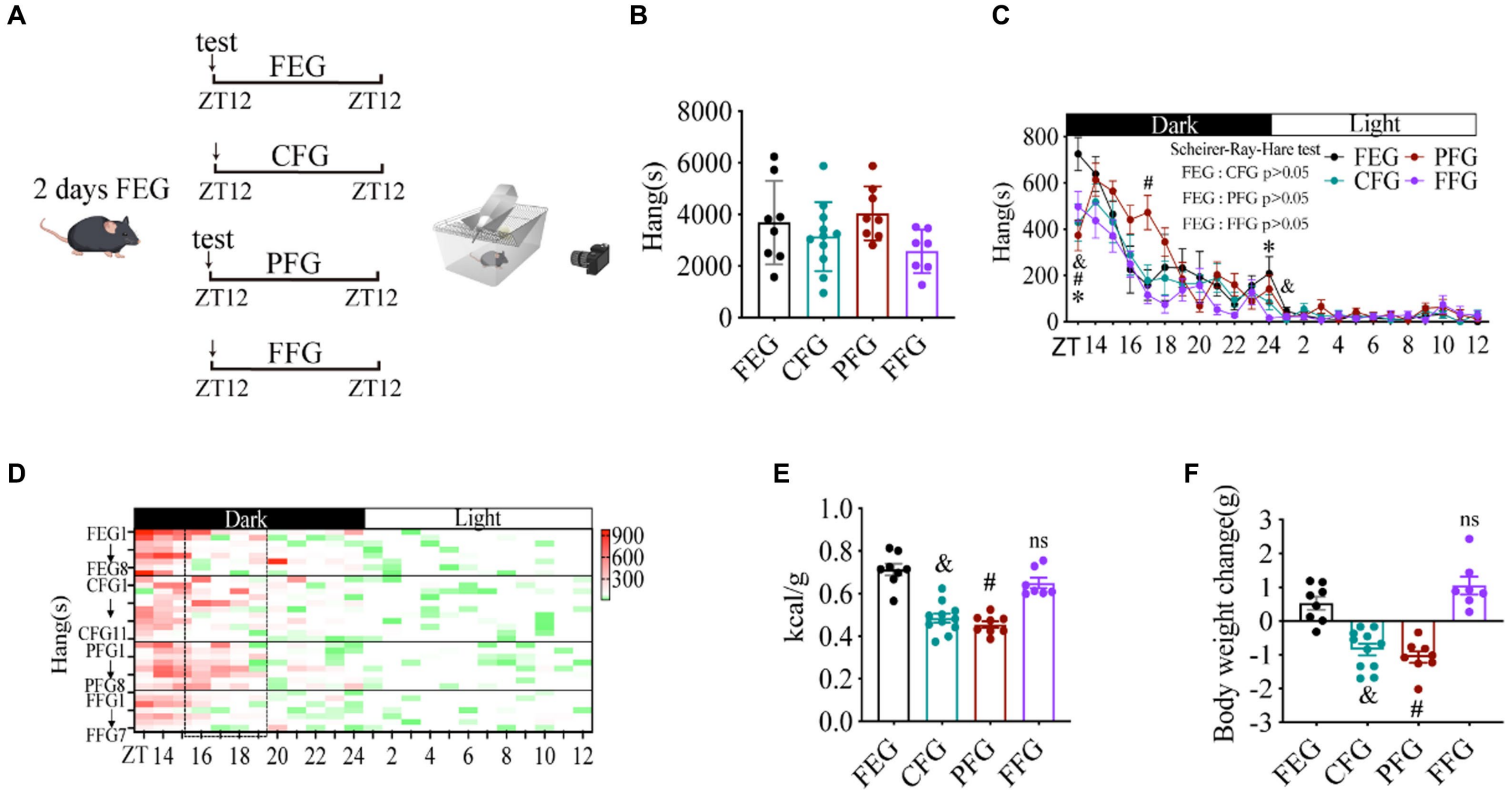
Habitual rhythm foraging behavior is dramatically increased by energy shortage instead of food component. **(A)** A diagram of experimental scheme. **(B)** Total time of hanging in 24 h. **(C)** Hanging time curve over 24 h. **(D)** Hanging time/h in 24 h for each mouse. **(E)** Calorie intake (kcal)/body weight (g) in different gel-fed mice. **(F)** Body weight change in different gel-fed mice. 10 weeks C57BL/6 J male mice were divided into four groups, *n* = 8:11:8:7. Data are represented as mean ± SEM. ^&^ represents the significance between FEG and CFG mice. ^#^ represents the significance between FEG and PFG mice. ^*^ represents the significance between FEG and FFG mice. ^&^*p* < 0.05, ^#^*p* < 0.05, ^*^p < 0.05. Data in histograms were analyzed using One-way ANOVA, and data in panel c were analyzed using the Scheirer-Ray-Hare test. ZT, zeitgeber time. FEG, full-energy gel. CFG, carbohydrate-free gel; PFG, protein-free gel; FFG, fat-free gel.

Since the energy source in food mainly derives from carbohydrate, fat, and protein (35), we next examined whether carbohydrate, fat, or protein plays an important role in the increase of foraging behavior induced by calorie content shortage. After fed with FEG for 2 days, carbohydrate-free gel (CFG), fat-free gel (FFG), protein-free gel (PFG), and FEG as control were used to feed mice, and all types of animal activity were monitored (Figure 4A). Total distance (Supplementary Figures S5A–C), activity time (Supplementary Figures S5D–F) and foraging behavior (Figures 4B–D) of mice were similar between CFG, PFG, FFG, and FEG groups (Scheirer-Ray-Hare test, *p* > 0.05), suggesting that the habitual rhythm foraging behavior was dramatically increased by energy deficiency rather than individual food components.

Next, we found that the deficiency of carbohydrate and protein reduced the calorie intake and body weight gain of mice (Figures 4E,F), suggesting that energy shortage need to reach a threshold for promoting foraging behavior and the small amounts of energy deficits may not have a significant effect. Indeed, protein deficiency increased the foraging behavior of mice at only one time point of ZT17 (Figure 4C). In contrast, the calorie intake and body weight gain of mice were similar between FFG and FEG groups (Figures 4E,F). Consistently, rearing (Supplementary Figures S5G–I) and grooming (Supplementary Figures S5M–O) were also not altered significantly (Scheirer-Ray-Hare test, *p* > 0.05). However, deficiency of fat increased the sniffing (Supplementary Figures S5J–L) and digging (Supplementary Figures S5P–R) behaviors (Scheirer-Ray-Hare test, *p* < 0.05), suggesting that fat was involved in the regulation of these behaviors.

**Figure 5:**
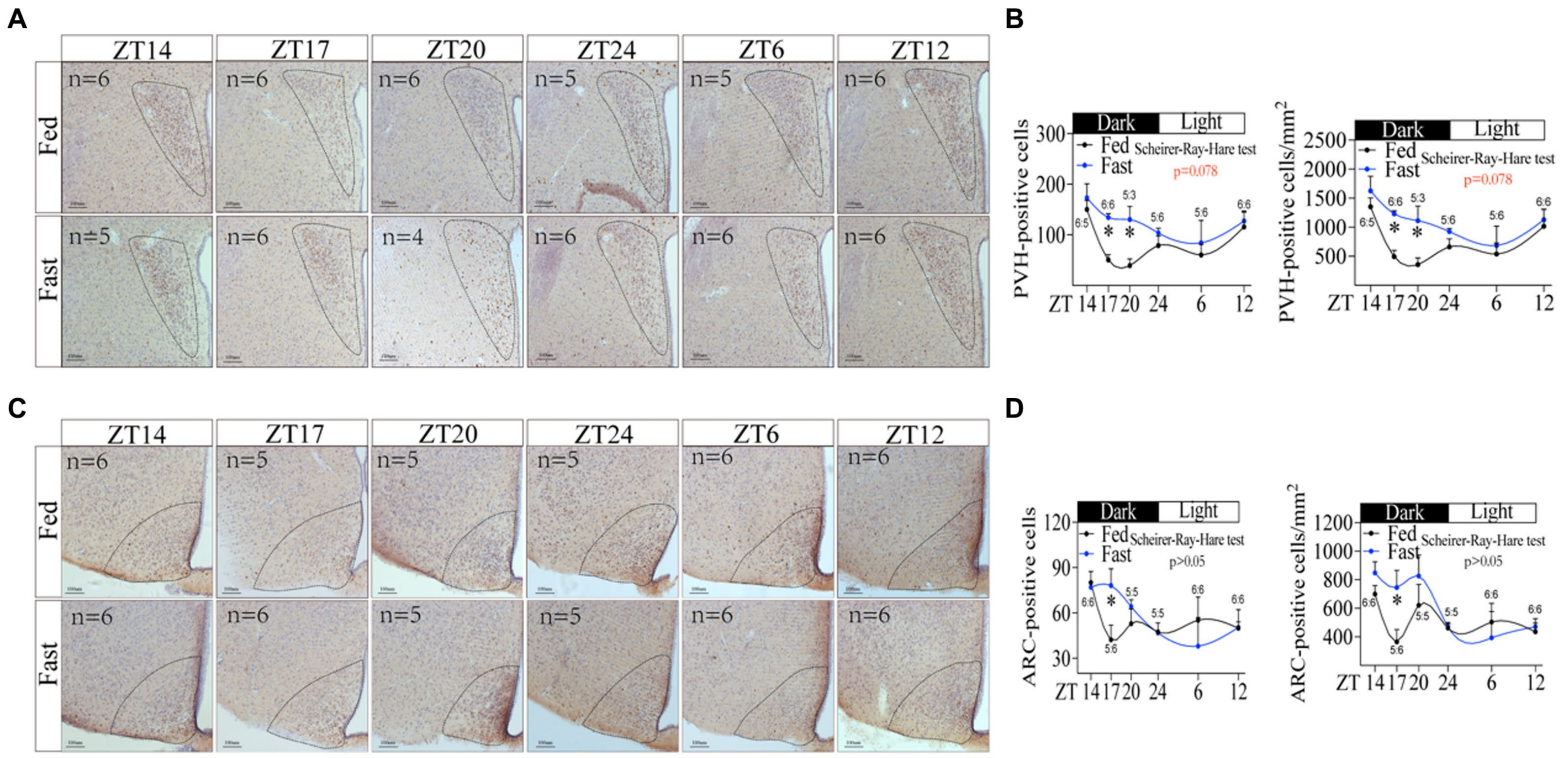
Neuronal activity in hypothalamic PVH is associated with the increasing rhythmic foraging behavior induced by energy deficiency. **(A)** Immunohistochemical staining of c-fos in PVH at different time points. **(B)** The number of c-fos positive cells and the density of c-fos positive cells in PVH. **(C)** Immunohistochemical staining of c-fos in ARC at different time points. **(D)** The number of c-fos positive cells and the density of c-fos positive cells in ARC. “n” numerical ratios in figures represent the number of mice per group at each time point. Only one brain slice at the corresponding location was selected for each mouse. PVH, ARC. Data were represented as mean ± SEM, ^*^p < 0.05. Data were analyzed using the Scheirer-Ray-Hare test. ZT, zeitgeber time.

Since hypothalamus is the center of regulating energy balance and feeding behavior (36, 37), we next examined whether hypothalamus is associated with the increase in rhythmic foraging behavior induced by energy deficiency. Using immunohistochemistry staining for c-fos, we assessed the activity of hypothalamic neurons in POA, SCN, ARC, PVH, DMH, VMH, and LH at different time points, respectively. The number and density of c-fos positive neurons in PVH were significantly altered (Figures 5A,B. Scheirer-Ray-Hare test *p* = 0.078). In particular, the number and density of positive cells in PVH were increased at night (Figure 5B), which was similar in the rhythmic character of the increased foraging behavior (Figure 1M). The c-fos expression in ARC was also increased during the night, but slightly reduced during the daytime in fasted mice compared to free-fed mice (Figures 5C,D). By contrast, fasting did not change the oscillation of c-fos expression in DMH, LH, VMH, and SCN (Supplementary Figures S6A–H), but disrupted the rhythmic oscillation of c-fos expression in POA (Supplementary Figures S6I,J). Notably, PVH is one of the most important autonomic control centers in the brain, which integrates endocrine, autonomic, and somatomotor systems from ARC, DMH, and VMH to influence feeding-related behavior (38). Thus, based on the rhythm and change of both c-fos expression and foraging behavior, PVH was possibly associated with the increased foraging behavior induced by energy deficiency.

**Figure 6:**
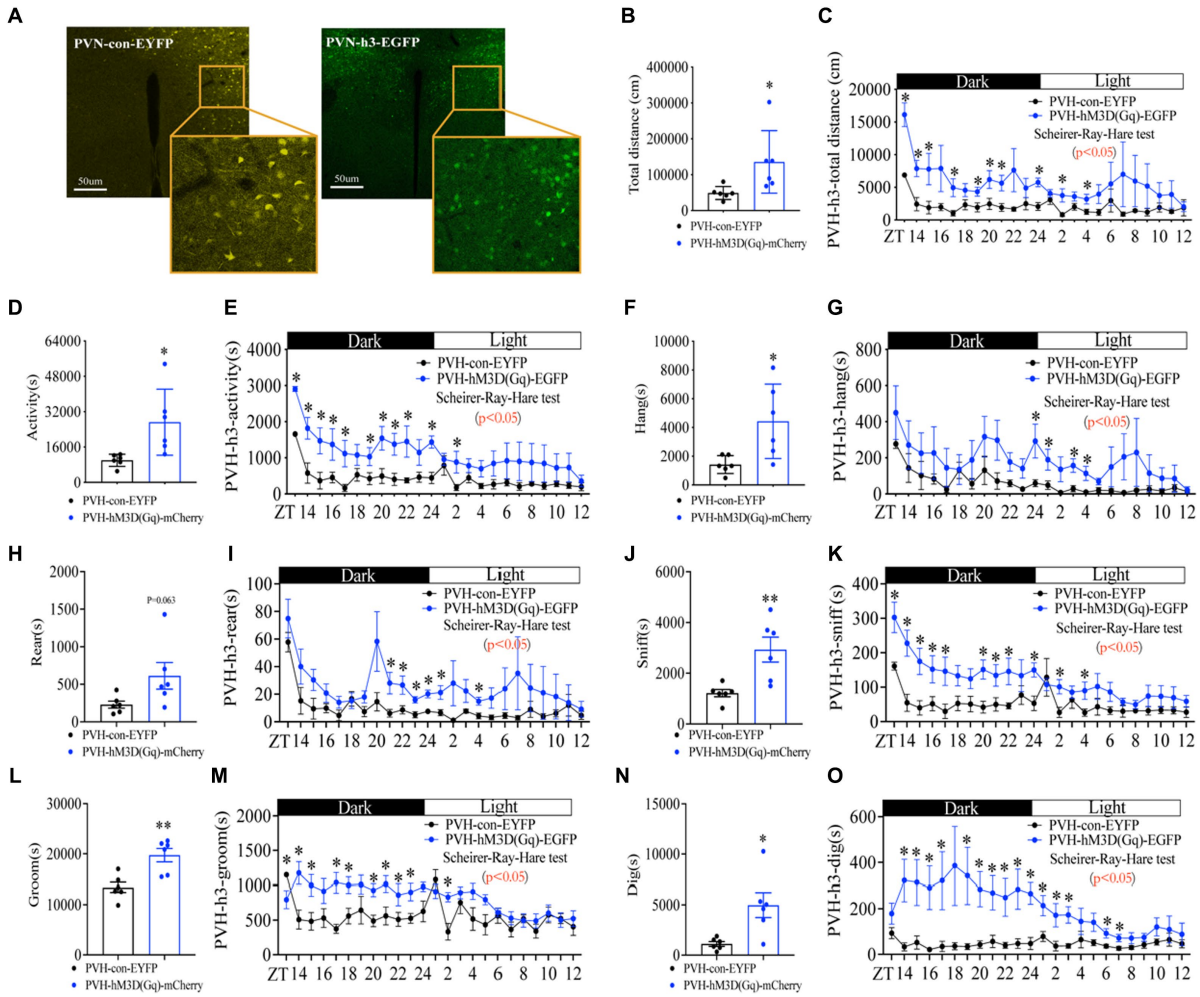
Activating neurons in PVH enhances the amplitude of foraging behavior. **(A)** hM3D(Gq) and control AAV2/9 expression in PVH. **(B)** Total distance in 24 h. **(C)** Distance curve over 24 h. **(D)** Total activity time in 24 h. **(E)** Activity time curve over 24 h. **(F)** Total hanging in 24 h. **(G)** Hanging curve over 24 h. **(H)** Total rearing in 24 h. **(I)** Rearing curve over 24 h. **(J)** Total sniffing in 24 h. **(K)** Sniffing curve over 24 h. **(L)** Total grooming in 24 h. **(M)** Grooming curve over 24 h. **(N)** Total digging in 24 h. **(O)** Digging curve over 24 h. 10 weeks C57BL/6 J male mice were injected with AAV2/9, and experiments were performed 3–4 weeks after virus injection, *n* = 6:6. Data are represented as mean ± SEM, ^*^p < 0.05, ^**^p < 0.01. Data in histograms were analyzed using an unpaired *t*-test, and other data were analyzed using the Scheirer-Ray-Hare test. ZT, zeitgeber time.

Next, we asked whether the neural activity in PVH neurons is responsible for the increase in foraging behavior. To address this issue, we used a chemogenetic approach to activate neuronal activity in PVH by expressing activated DREADDs (Gq hM3D) (Supplementary Figure S7A; Figure 6A). After CNO injection (1 mg/kg, 5 min before ZT12), PVH^hM3D(Gq)^ mice displayed a significant increase in total distance (Figures 6B,C and Supplementary Figure S7B), general activity (Figures 6D,E and Supplementary Figure S7C) and foraging behavior (Figures 6F,G and Supplementary Figure S7D) during fed with FEG, suggesting that the neuron activity in PVH was sufficient to promote the increase of foraging behavior. However, activating neurons in PVH not only increased the foraging behavior, but also increased rearing (Figures 6H,I and Supplementary Figure S7E), sniffing (Figures 6J,K and Supplementary Figure S7F), grooming (Figures 6L,M and Supplementary Figure S7G), and digging (Figures 6N,O and Supplementary Figure S7H) behaviors. Taken together, PVH neuronal activity is sufficient for eliciting foraging behavior.

**Figure 7:**
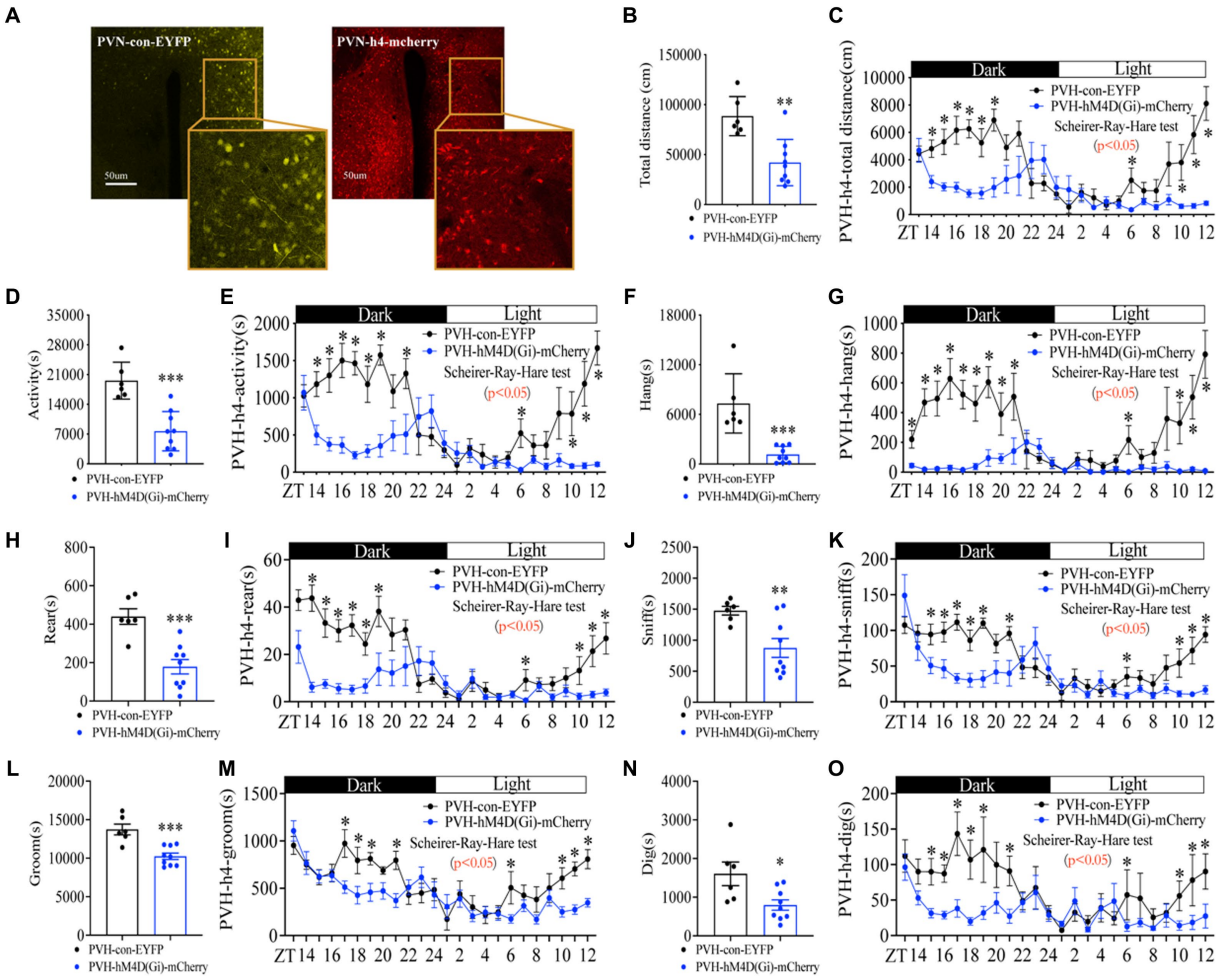
Inactivating neurons in PVH attenuates the amplitude of foraging behavior and disrupts the rhythm of foraging behavior. **(A)** hM4D(Gi) and control AAV2/9 expression in PVH. **(B)** Total distance in 24 h. **(C)** Distance curve over 24 h. **(D)** Total activity time in 24 h. **(E)** Activity time curve over 24 h. **(F)** Total hanging in 24 h. **(G)** Hanging curve over 24 h. **(H)** Total rearing in 24 h. **(I)** Rearing curve over 24 h. **(J)** Total sniffing in 24 h. **(K)** Sniffing curve over 24 h. **(L)** Total grooming in 24 h. **(M)** Grooming curve over 24 h. **(N)** Total digging in 24 h. **(O)** Digging curve over 24 h. 10 weeks C57BL/6 J male mice were injected with AAV2/9, and experiments were performed 3–4 weeks after virus injection, *n* = 6:6. Data are represented as mean ± SEM, ^*^p < 0.05, ^**^p < 0.01, ^***^p < 0.001. Data in histograms were analyzed using an unpaired *t*-test, and other data were analyzed using the Scheirer-Ray-Hare test. ZT, zeitgeber time.

Next, we determined whether neuron activity in PVH is required for the increase of foraging behavior induced by energy deficiency. PVH neuron activity was inhibited by expressing the inhibitory DREADDs (Gi hM4D) in PVH (Supplementary Figure S8A; Figure 7A). After CNO injection (1 mg/kg, 5 min before ZT12), PVH^hM4D(Gi)^ mice displayed a significant decrease in total distance (Figures 7B,C and Supplementary Figure S8B), general activity (Figures 7D,E and Supplementary Figure S8C), and foraging behavior (Figures 7F,G and Supplementary Figure S8D) during fed with NEG, suggesting that the neuron activity in PVH is required to promote the increase of foraging behavior. Notably, inactivated PVH neuron activity impaired the rhythm of foraging behavior and suppressed almost all of foraging behavior (Figure 7G), suggesting that the neuron activity in PVH is required to sustain the foraging behavior. Consistently, PVH^hM4D(Gi)^ mice displayed a dramatic decrease in rearing (Figures 7H,I and Supplementary Figure S8E), sniffing (Figures 7J,K and Supplementary Figure S8F), grooming (Figures 7L,M and Supplementary Figure S8G), and digging (Figures 7N,O and Supplementary Figure S8H) behaviors. Taken together, neuron activity in PVH is required for the increased foraging behavior induced by energy deficiency.

## Discussion

The foraging behavior is similar to feeding, reproduction, metabolism, and other physiological processes, which are essential for life (39). In this report, we demonstrate that food cues and energy shortage, rather than macronutrients deficiency, potentiate the rhythmic foraging behavior. This is the first to report that the hanging behavior of fasted mice reflects the nature or characterize of foraging behavior, and is regulated by the activity of PVH neurons.

In humans, dysregulated foraging behavior leads to a variety of pathological manifestations, such as anorexia nervosa (40), binge-eating disorder (41), night-eating syndrome (41), sleep-related eating disorder (42), and obesity (41, 43). Therefore, the study of foraging behavior is crucial for human health. Consistent with the results in animals, a study of human foraging behavior found that people tend to seek out high-calorie foods in supermarkets (44). In addition to calorie-seeking, human foraging behavior is also affected by environment and cultural rituals (45). Thus, normative studies of human foraging behavior are very difficult. In contrast, laboratory animal models are good tools for studying foraging behavior, which will be beneficial in understanding human foraging behavior.

In laboratory mice, hanging behavior is not a unique manifestation of a single function. In previous studies, researchers measured pain and motor neuron function by monitoring hanging behavior in caged mice (46, 47). In this report, we broke the behavior of fasted mice into various fine behaviors and found that only the hanging behavior was increased, similar to the increased total activity (Figure 1), suggesting that the hanging behavior reflects the nature of foraging behavior in fasted mice. Furthermore, mice have adopted the perception of food on the lid of the cage, food deprivation will elicit the hanging behavior when mice attach themselves to the cage lid with their legs off the ground to search for food. Of note, other species or genera of animals, such as bats, flying foxes, and arboreal mammals, show that hanging behavior is a typical action of searching for food (48–50). Although these species of animals are not equal to caged mice, most animal behaviors are similar or equivalent in different animal species. Thus, laboratory mice can be used as a model to study foraging behavior by monitoring the hanging behavior.

Our observations suggest that energy status or hunger signals are critical regulators for foraging behavior (Figures 1M, 2C). However, the partial energy deficit caused by inadequate CFG and PFG intake (Figure 4E) did not cause significant increases in foraging behavior (Figure 4B), suggesting that sufficient energy deficiency is required to motivate foraging behavior. Sufficient energy deficiency is a potent motivator of foraging behavior, which is modulated by evolutionary pressures and governed by hormonal (51), neural (52), and cognitive systems (53). Because the innate drive to restore energy balance is robust (54–56), small energy deficits may not be sufficient to increase foraging behavior.

Grooming and digging were decreased in mice under fasting conditions compared with fed mice (Supplementary Figures S1E–H), while mice fed with NEG showed unaltered grooming and digging behaviors compared with mice fed by FEG (Supplementary Figures S2M–R), suggesting that food cues were involved in regulating grooming and digging. Indeed, grooming and digging behaviors in many animals, especially rodents, are often related to food cues. For example, when food cues cannot predict a certain outcome (e.g., unpredictable food delivery), mice might increase grooming as a way to release stress (57). In addition, food cues might trigger an innate digging response in certain animals (e.g., the scent of food source) (58). In contrast to food deprivation, the presence of NEG provides conditions or choices for mice to dig up or bury food (gel), although NEG is energy-free.

To rule out the interference in the physiological activities of mice during 24 h of continuous Home-Cage monitoring, we administered a single injection of CNO to mice 5 min before the Home-Cage monitoring. Despite the short plasma half-life of CNO in mice (59, 60), the biological effects of CNO can also last as long as 6–10 h (59, 61). Thus, a single injection is sufficient to observe changes in foraging behavior at night in our experiments.

The habitual rhythm of foraging behavior strikingly disappeared when inactivated neurons in PVH (Figure 7G). One possible reason is that the biological effects of CNO can be maintained for over 10 h. Another possibility is that the CNO injection continuously inactivates neuronal activity by binding to DREADDs (24), which masks or disrupts the habitual rhythm of neuronal activity. Of note, extensive regulation of PVH neuronal activity altered all types of behaviors (Figures 6, 7), suggesting that multiple types of neurons in PVH play differential roles in regulating foraging behavior. Indeed, besides feeding behavior, the PVH is also a critical region of the brain involved in other behaviors. For example, activation of the PVH results in the release of corticotropin-releasing hormone (CRH), which in turn triggers the pituitary to release adrenocorticotropic hormone (ACTH) to release glucocorticoids like cortisol, which helps the body respond to stress (62). PVH is also involved in the secretion of gonadotropin-releasing hormone (GnRH), which regulates reproductive hormones and associated behaviors (63). In addition, PVH has been implicated in various social behaviors, possibly due to its role in releasing oxytocin, which is often called the “love hormone” because of its role in pair bonding, social recognition, and other prosocial behaviors (64).

This work is the first to discover the equivalent link between hanging behavior and foraging behavior in caged mice and provides a laboratory mouse model for studying foraging behavior. However, there are some limitations in this study. First, the chemogenetic approach cannot be used to control the rhythm of PVH neuronal activity in mice. Second, the different types of neurons in PVH were not distinguished or identified. Therefore, further experiments are required for the identification of the specific neurons in PVH for regulating rhythmic foraging behavior.

Understanding the foraging behavior has significance in basic biological processes and provides potentially practical applications in managing human eating behavior, preventing eating-related disorders, and developing personalized dietary styles. Considering the effect of energy status and food cues on foraging behavior, researchers and healthcare professionals can design healthier food choices to intervene in overeating behavior and malnutrition.

## Conclusion

In summary, we conclude that hanging behavior reflects the properties of foraging behavior in fasted mice, and neuronal activity in PVH is required for triggering and maintaining the foraging behavior. This work will provide fresh insights into the cellular mechanisms for foraging behavior and a better therapeutic target for eating-related disorders.

## Data availability statement

The original contributions presented in the study are included in the article/Supplementary material, further inquiries can be directed to the corresponding author.

## Ethics statement

The animal study was approved by Institutional Animal Care and Use Committee at Shandong Provincial Hospital. The study was conducted in accordance with the local legislation and institutional requirements.

## Author contributions

SW: Conceptualization, Data curation, Formal analysis, Investigation, Methodology, Project administration, Software, Supervision, Validation, Writing – original draft, Writing – review & editing. JW: Writing – review & editing. YX: Software, Writing – review & editing. ZZ: Formal analysis, Software, Writing – review & editing. XJ: Writing – review & editing. YL: Writing – review & editing. YG: Writing – review & editing. HZ: Writing – review & editing. LP: Writing – review & editing. DL: Writing – review & editing. ML: Writing – review & editing. WB: Writing – review & editing, Funding acquisition. QG: Writing – review & editing. ZH: Conceptualization, Data curation, Formal analysis, Funding acquisition, Investigation, Methodology, Project administration, Software, Supervision, Validation, Writing – review & editing.
