# Supplementary material for: Energy deficiency promotes rhythmic foraging behavior by activating neurons in paraventricular hypothalamic nucleus
Source: Front Nutr. 2023 Oct 12;10:1278906. doi: 10.3389/fnut.2023.1278906 (PMC10600490; doi:10.3389/fnut.2023.1278906)
Supplement: Supplementary file 1 [file Data_Sheet_1.pdf]

Supplemental Figures

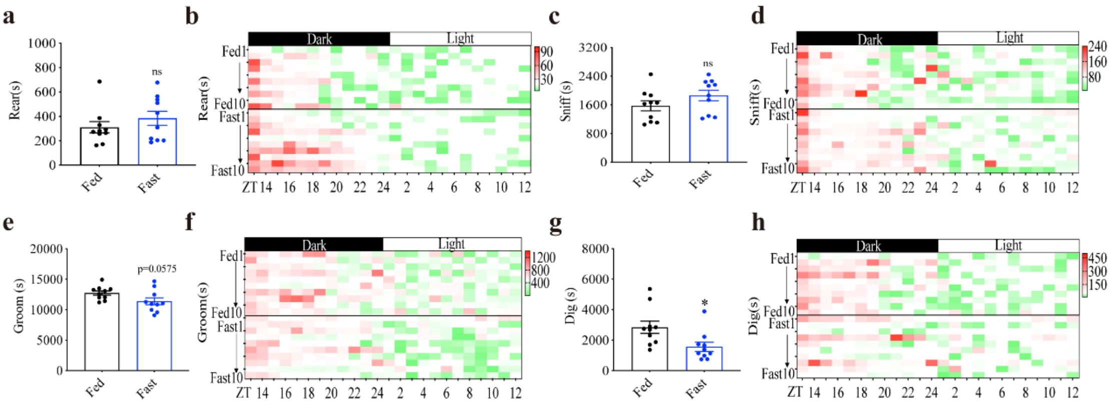

Figure S1. Behaviors in fed or fasted mice

a. Total time of rearing in 24 hours. **b**. Rearing time/hour in 24 hours for each mouse. **c**. Total time of sniffing in 24 hours. **d**. Sniffing time/hour in 24 hours for each mouse. **e**. Total time of grooming in 24 hours. **f**. Grooming time/hour in 24 hours for each mouse. **g**. Total time of digging in 24 hours. **h**. Digging time/hour in 24 hours for each mouse. 10 weeks C57BL/6J male mice fed by chow diet or fasted, n=10:10. Data are represented as mean ± SEM. *p<0.05, ns represents not significant. Data in the histogram were analyzed using an unpaired t-test. ZT: zeitgeber time.

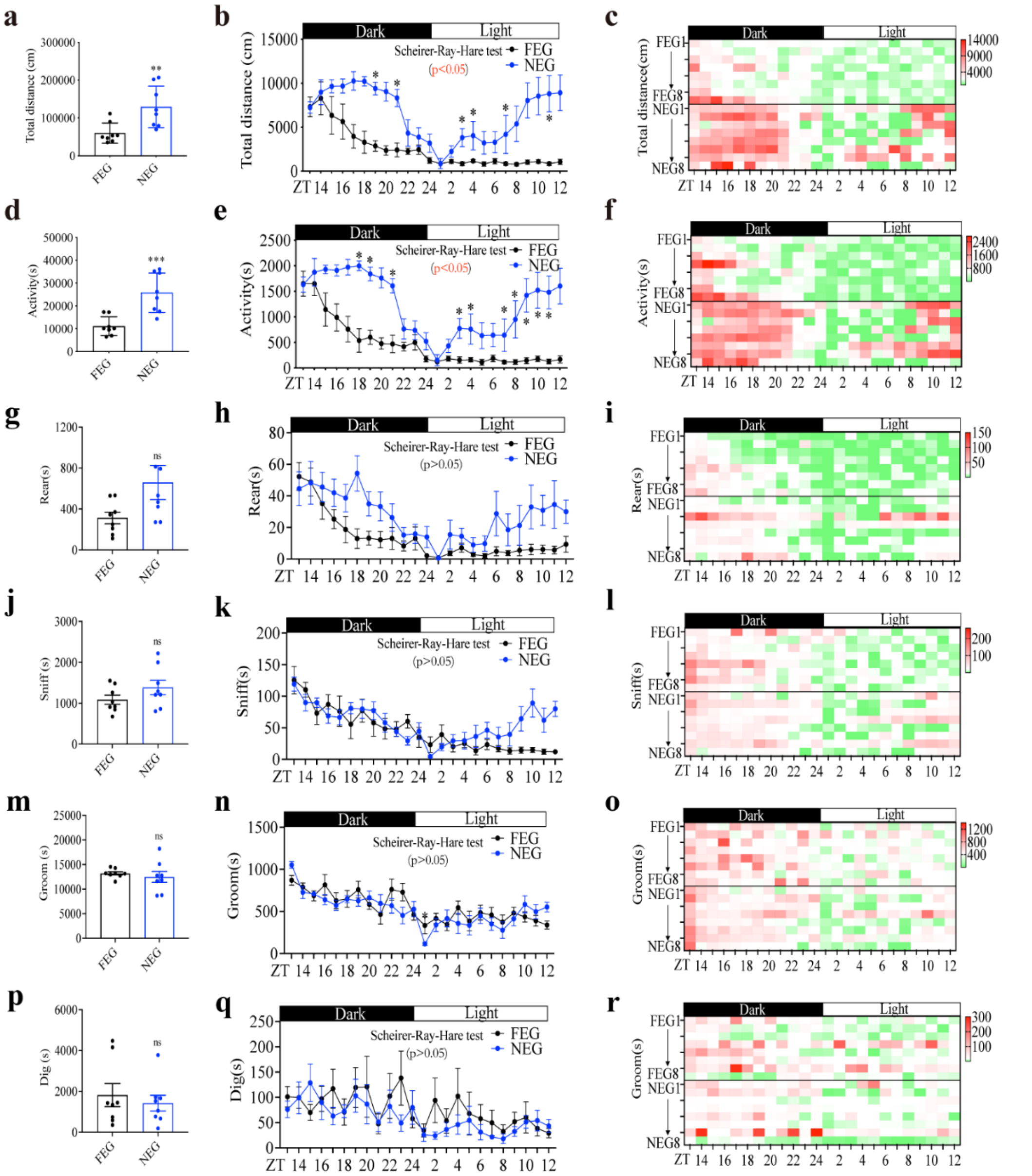

Figure S2. Behaviors of mice fed by FEG and NEG

a. Total distance in 24 hours. **b**. Distance curve per hour over 24 hours. **c**. Distance/hour in 24 hours for each mouse. **d**. Total time of activity in 24 hours. **e**. Activity curve per hour over 24 hours. **f**. Activity/hour in 24 hours for each mouse. **g**. Total rearing time in 24 hours. **h**. Rearing curve per hour over 24 hours. **i**. Rearing/hour in 24 hours for each mouse. **j**. Total sniffing time in 24 hours. **k**. Sniffing curve per hour over 24 hours. **l**. Sniffing/hour in 24 hours for each mouse. **m**. Total grooming time in 24 hours, **n**. Grooming curve per hour over 24 hours. **o**. Grooming/hour in 24 hours for each mouse. **p**. Total digging time in 24 hours. **q**. digging curve per hour over 24 hours. **r**.

Digging/hour in 24 hours for each mouse. Data were represented as mean ± SEM, *p<0.05, **p<0.01, ***p<0.001. 12 weeks C57BL/6J male mice fed with FEG or NEG, n=8:8. Data in histograms were analyzed using an unpaired t-test, data in panel n was analyzed using two-way repeated measurements ANOVA, and other data were analyzed using Scheirer-Ray-Hare test. ZT: zeitgeber time. FEG: full-energy gel. NEG: non-energy gel. ZT: zeitgeber time.

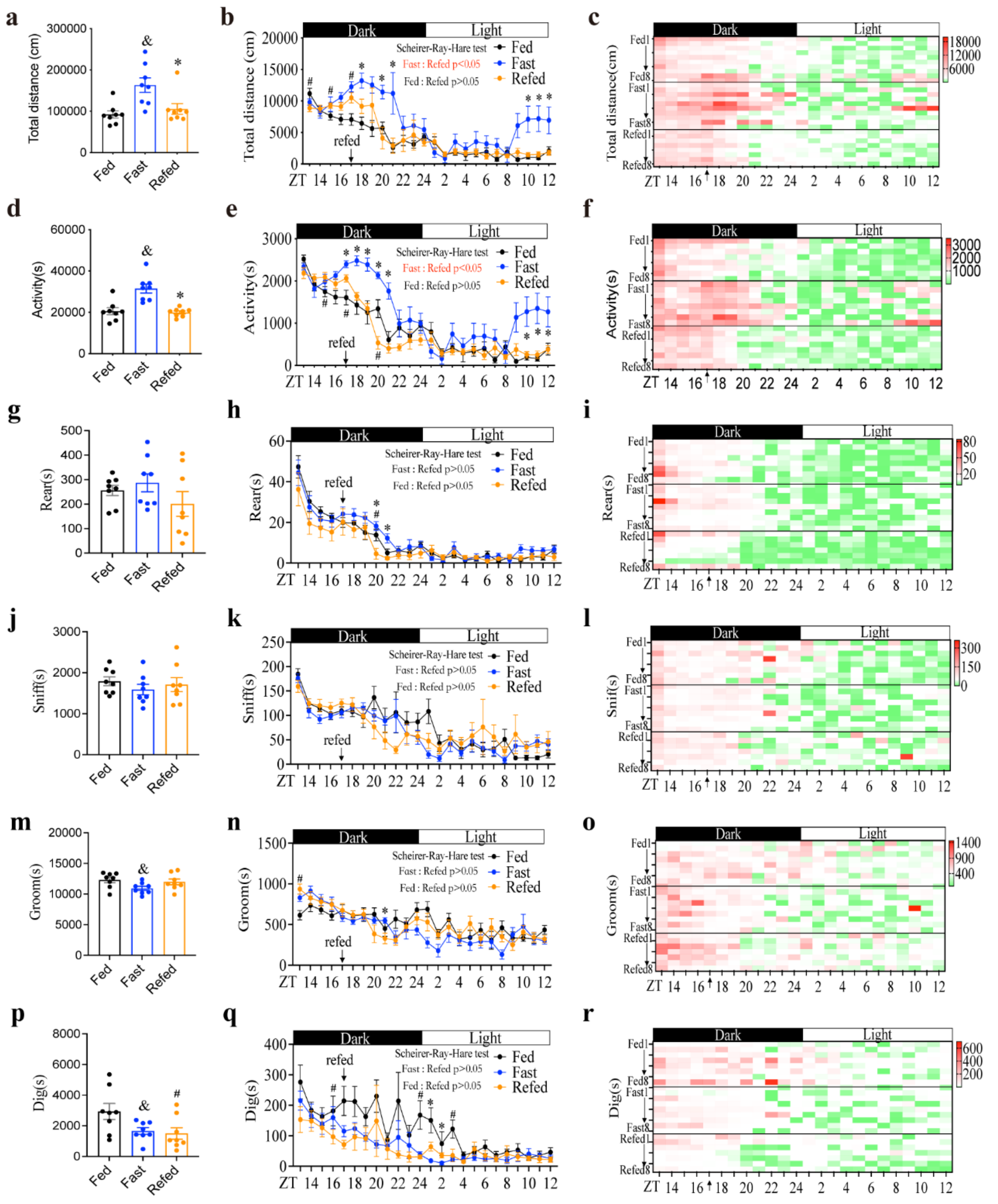

Figure S3. Behaviors in refed mice

a. Total distance in 24 hours. **b**. Distance curve over 24 hours. **c**. Distance/hour in 24 hours for each mouse. **d**. Total time of activity in 24 hours. **e**. Activity curve over 24 hours. **f**. Activity/hour in 24 hours for each mouse. **g**. Total rearing time in 24 hours, **h**. Rearing curve over 24 hours. **i**. Rearing/hour in 24 hours for each mouse. **j**. Total sniffing time in 24 hours. **k**. Sniffing curve over 24 hours. **l**. Sniffing/hour in 24 hours for each mouse. **m**. Total grooming time in 24 hours. **n**. Grooming curve over 24 hours. **o**. Grooming/hour in 24 hours for each mouse. **p**. Total digging time in 24 hours. **q**. Digging curve over 24 hours. **r**. Digging/hour in 24 hours for each mouse. Data were represented as mean ± SEM. $^{\&}$ represents the significance between fed and fasted mice. $^{\#}$ represents the significance between fed and refed mice. * represents the significance between fasted and refed mice. $^{\&}p<0.05$, $^{\#}p<0.05$, $^{*}p<0.05$. 10 weeks C57BL/6J male mice were subjected to this refed experiment, n=8:8:8. Data in histograms were analyzed using two-way repeated measurements ANOVA, and other data were analyzed using Scheirer-Ray-Hare test. ZT: zeitgeber time.

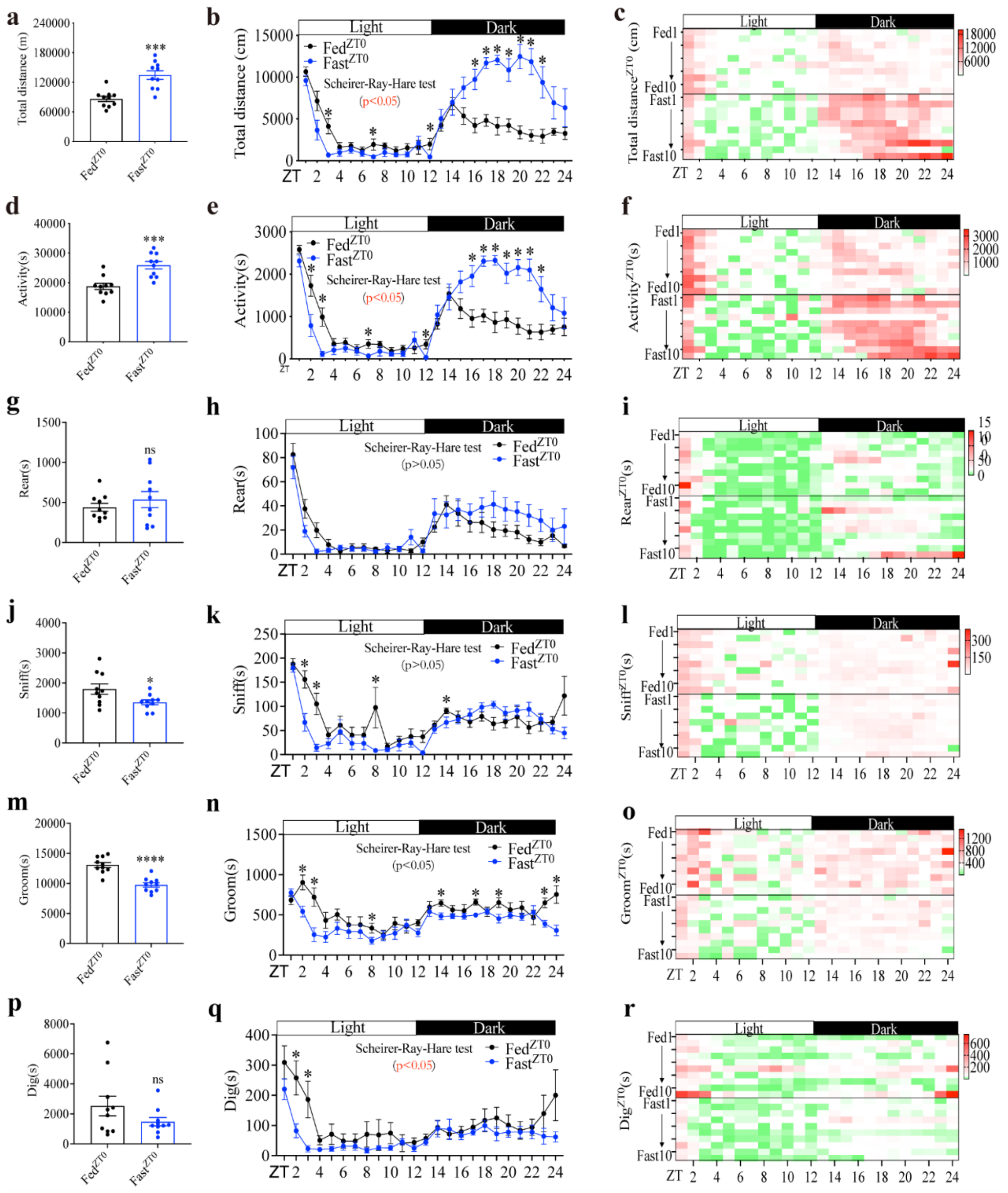

Figure S4. Behaviors in fed or fasted mice

a. Total distance in 24 hours. **b**. Distance curve over 24 hours. **c**. Distance/hour in 24 hours for each mouse. **d**. Total time of activity in 24 hours. **e**. Activity curve over 24 hours. **f**. Activity/hour in 24 hours for each mouse. **g**. Total rearing time in 24 hours. **h**. Rearing curve over 24 hours. **i**. Rearing/hour in 24 hours for each mouse. **j**. Total sniffing time in 24 hours. **k**. Sniffing curve over 24 hours. **l**. Sniffing/hour in 24 hours for each mouse. **m**. Total grooming time in 24 hours. **n**. Grooming curve over 24 hours.

o. Grooming/hour in 24 hours for each mouse. **p**. Total digging time in 24 hours. **q**. Digging curve over 24 hours. **r**. Digging/hour in 24 hours for each mouse. Data were represented as mean ± SEM. *p<0.05, ***p<0.001. 12 weeks C57BL/6J male mice fed with chow diet or fasted were monitored by Home-Cage system from ZT0, n=10:10. Data in histograms were analyzed using an unpaired t-test, and other data were analyzed using Scheirer-Ray-Hare test. ZT: zeitgeber time.

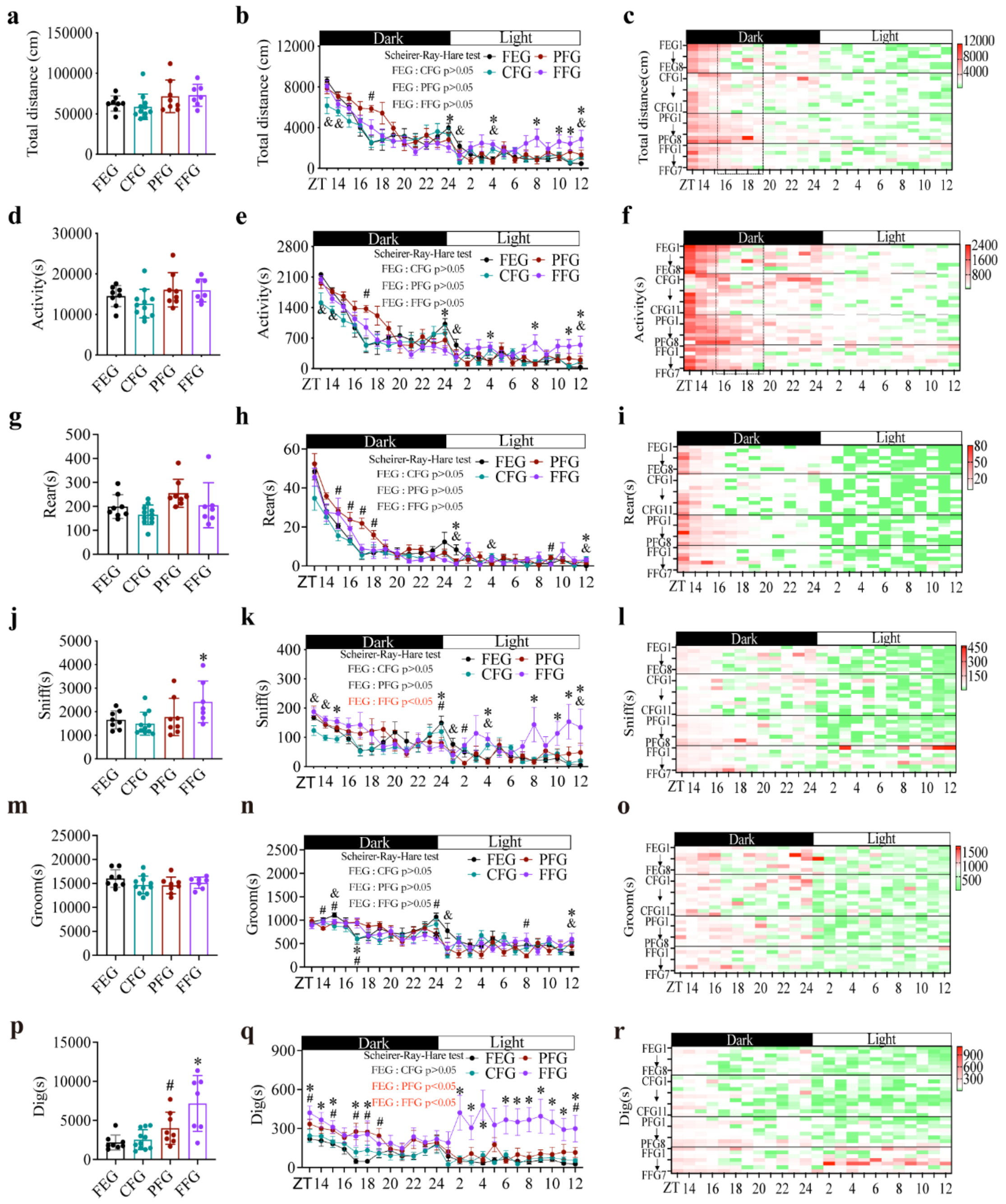

Figure S5. Behaviors in mice fed with different nutrients deficiency gel

a. Total distance in 24 hours. **b**. Distance curve over 24 hours. **c**. Distance/hour in 24 hours for each mouse. **d**. Total time of activity in 24 hours. **e**. Activity curve over 24 hours. **f**. Activity/hour in 24 hours for each mouse. **g**. Total rearing time in 24 hours, h. Rearing curve over 24 hours. **i**. Rearing/hour in 24 hours for each mouse. **j**. Total sniffing time in 24 hours. **k**. Sniffing curve over 24 hours, l. Sniffing/hour in 24 hours for each mouse. **m**. Total grooming time in 24 hours. **n**. Grooming curve over 24 hours. **o**. Grooming/hour in 24 hours for each mouse. **p**. Total digging time in 24 hours. **q**. Digging curve over 24 hours. **r**. Digging/hour in 24 hours for each mouse. 10 weeks C57BL/6J male mice were divided into four groups, n=8:11:8:7. Data were represented as mean ± SEM. $^{\&}$ represents the significance between FEG and CFG mice. $^{\#}$ represents the significance between FEG and PFG mice. * represents the significance between FEG and FFG mice. $^{\&}p<0.05$, $^{\#}p<0.05$, $^{*}p<0.05$. Data in histograms were analyzed using One-way ANOVA, and other data were analyzed using the Scheirer-Ray-Hare test. ZT: zeitgeber time. FEG: full-energy gel. CFG: carbohydrate-free gel. PFG: protein-free gel. FFG: fat-free gel.

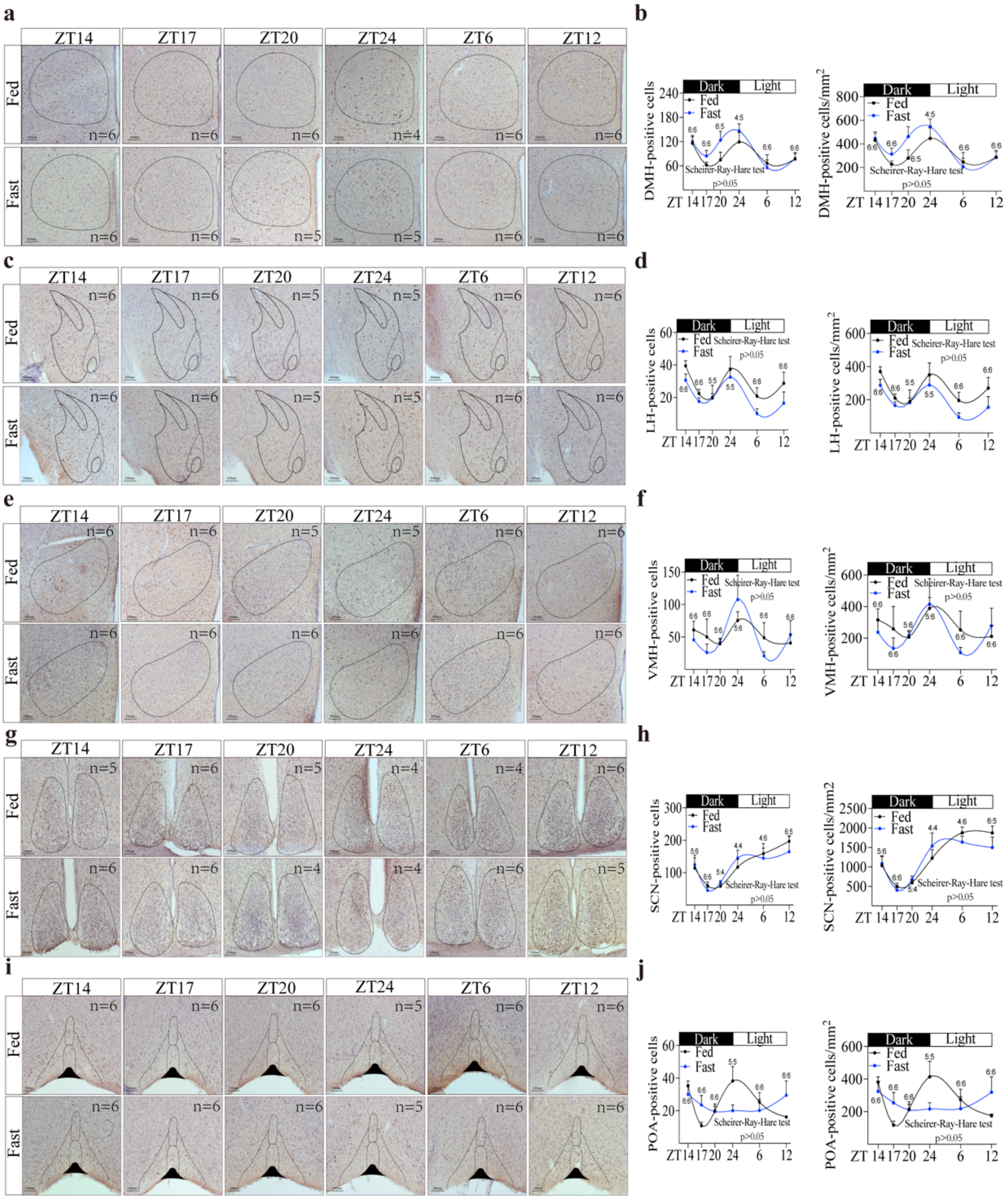

Figure S6. The expression of hypothalamic c-fos in mice

a. Immunohistochemical staining of c-fos in DMH at different time points. **b**. The number of c-fos positive cells and the density of positive cells in DMH. c. Immunohistochemical staining of c-fos in LH at different time points. **d**. The number of c-fos positive cells and the density of positive cells in LH. e. Immunohistochemical staining of c-fos in VMH at different time points. **f**. The number of c-fos positive cells and the density of positive cells in VMH. g. Immunohistochemical staining of c-fos in SCN at different time points. **h**. The number of c-fos positive cells and the density of

positive cells in SCN. i. Immunohistochemical staining of c-fos in POA at different time points. **j**. The number of c-fos positive cells and the density of positive cells in POA. “n” and numerical ratios in figures represent the number of mice per group at each time point, only one brain slice at the corresponding location was selected for each mouse. DMH, POA, VMH, LH, and SCN. Data were represented as mean ± SEM. Data were analyzed using the Scheirer-Ray-Hare test. ZT: zeitgeber time.

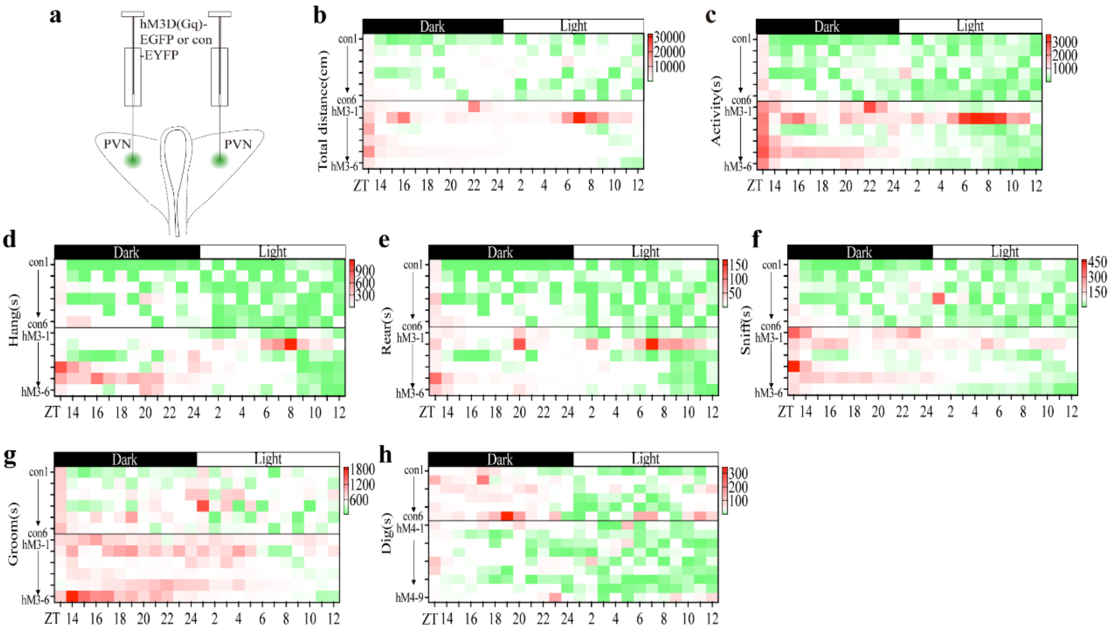

Figure S7. Behaviors in hM3D(Gq) AAV2/9 injection mice

a. A diagram of experimental scheme(hM3D(Gq) or control AAV2/9 expression in PVH). **b**. Distance/hour in 24 hours for each mouse in $PVH^{hM3D(Gq)}$ mice. **c**. Activity time/hour in 24 hours for each mouse in $PVH^{hM3D(Gq)}$ mice. **d**. Hanging/hour in 24 hours for each mouse in $PVH^{hM3D(Gq)}$ mice. **e**. Rearing/hour in 24 hours for each mouse in $PVH^{hM3D(Gq)}$ mice. **f**. Sniffing/hour in 24 hours for each mouse in $PVH^{hM3D(Gq)}$ mice. **g**. Grooming/hour in 24 hours for each mouse in $PVH^{hM3D(Gq)}$ mice. **h**. Digging/hour in 24 hours for each mouse in $PVH^{hM3D(Gq)}$ mice. ZT: zeitgeber time.

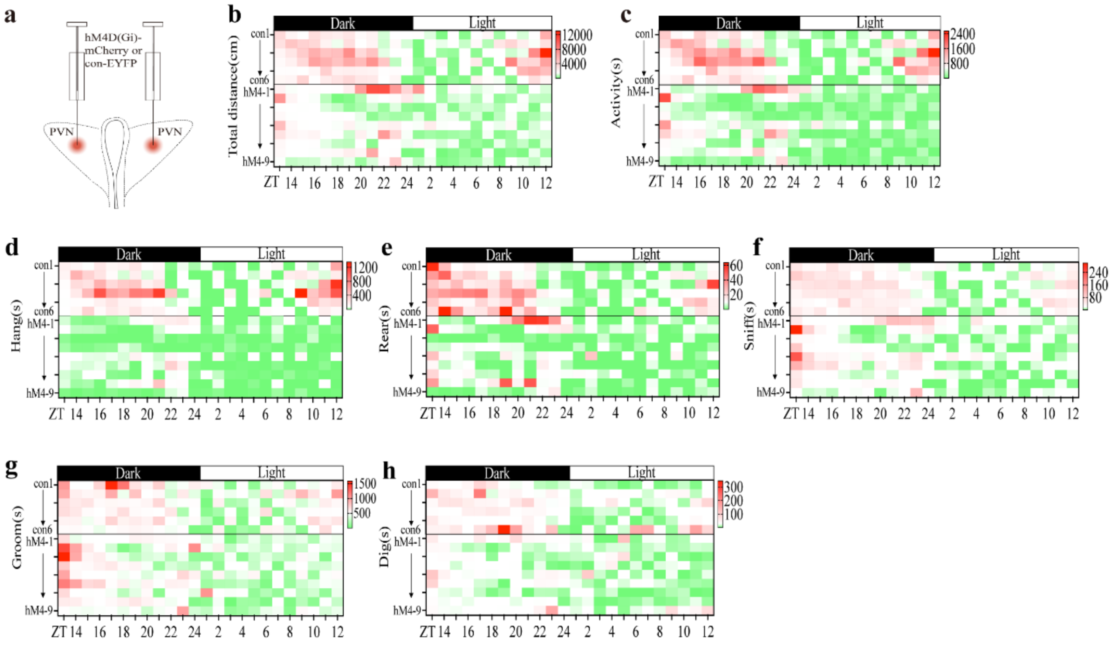

Figure S8. Behaviors in hM4D(Gi) AAV2/9 injection mice

a. A diagram of experimental scheme(hM4D(Gi) or control AAV2/9 expression in PVH). **b**. distance/hour in 24 hours for each mouse in PVH$^{hM4D(Gi)}$ mice. **c**. activity time/hour in 24 hours for each mouse in PVH$^{hM4D(Gi)}$ mice. **d**. hanging/hour in 24 hours for each mouse in PVH$^{hM4D(Gi)}$ mice. **e**. rearing/hour in 24 hours for each mouse in PVH$^{hM4D(Gi)}$ mice. **f**. sniffing/hour in 24 hours for each mouse in P PVH$^{hM4D(Gi)}$ mice. **g**. grooming/hour in 24 hours for each mouse in PVH$^{hM4D(Gi)}$ mice. **h**. digging/hour in 24 hours for each mouse in PVH$^{hM4D(Gi)}$ mice. ZT: zeitgeber time.
